# Regulatory effect of calcineurin inhibitor, tacrolimus, on IL-6/sIL-6R-mediated RANKL expression through JAK2-STAT3-SOCS3 signaling pathway in fibroblast-like synoviocytes

**DOI:** 10.1186/ar4162

**Published:** 2013-02-13

**Authors:** Jung-Yoon Choe, Ki-Yeun Park, Sung-Hoon Park, Sang-Il Lee, Seong-Kyu Kim

**Affiliations:** 1 Division of Rheumatology, Department of Internal Medicine, Catholic University of Daegu School of Medicine, 3056-6 Daemyung 4-Dong, Namgu, Daegu 705-718, Republic of Korea; 2 Arthritis and Autoimmunity Research Center, Catholic University of Daegu School of Medicine, 3056-6 Daemyung 4-Dong, Namgu, Daegu 705-718, Republic of Korea; 3 Department of Internal Medicine and Institute of Health Science, Gyeongsang National University School of Medicine, 92 Chilam-Dong, Jinju, Gyeongnam 660-702 Republic of Korea

## Abstract

**Introduction:**

This study investigated whether the calcineurin inhibitor, tacrolimus, suppresses receptor activator of NF-κB ligand (RANKL) expression in fibroblast-like synoviocytes (FLS) through regulation of IL-6/Janus activated kinase (JAK2)/signal transducer and activator of transcription-3 (STAT3) and suppressor of cytokine signaling (SOCS3) signaling.

**Methods:**

The expression of RANKL, JAK2, STAT3, and SOCS3 proteins was assessed by western blot analysis, real-time PCR and ELISA in IL-6 combined with soluble IL-6 receptor (sIL-6R)-stimulated rheumatoid arthritis (RA)-FLS with or without tacrolimus treatment. The effects of tacrolimus on synovial inflammation and bone erosion were assessed using mice with arthritis induced by K/BxN serum. Immunofluorescent staining was performed to identify the effect of tacrolimus on RANKL and SOCS3. The tartrate-resistant acid phosphatase staining assay was performed to assess the effect of tacrolimus on osteoclast differentiation.

**Results:**

We found that RANKL expression in RA FLS is regulated by the IL-6/sIL-6R/JAK2/STAT3/SOCS3 pathway. Inhibitory effects of tacrolimus on RANKL expression in a serum-induced arthritis mice model were identified. Tacrolimus inhibits RANKL expression in IL-6/sIL-6R-stimulated FLS by suppressing STAT3. Among negative regulators of the JAK/STAT pathway, such as CIS1, SOCS1, and SOCS3, only SOCS3 is significantly induced by tacrolimus. As compared to dexamethasone and methotrexate, tacrolimus more potently suppresses RANKL expression in FLS. By up-regulating SOCS3, tacrolimus down-regulates activation of the JAK-STAT pathway by IL-6/sIL-6R trans-signaling, thus decreasing RANKL expression in FLS.

**Conclusions:**

These data suggest that tacrolimus might affect the RANKL expression in IL-6 stimulated FLS through STAT3 suppression, together with up-regulation of SOCS3.

## Introduction

Receptor activator of NF-κB ligand (RANKL) is a transmembrane protein of the TNF superfamily, which is an important molecule in bone metabolism [1]. RANKL, together with macrophage colony-stimulating factor (M-CSF), is an essential molecule in osteoclast formation through its role in the differentiation of osteoclast precursor cells into multinuclear osteoclast-like cells with bone resorbing activity. RANKL produced by infiltrating active T cells and macrophages was highly detectable in the synovial tissues of subjects with active rheumatoid arthritis (RA) [2,3]. Fibroblast-like synoviocytes (FLS), which are stimulated by IL-6, TNF-α and IL-17, are crucial cells that produce RANKL in the inflammatory joints of patients with RA [3-5]. These findings suggest that RANKL has an important role in bone resorption and loss, with FLS acting as a major producer of RANKL in RA.

The IL-6 and IL-6R complex leads to homodimerization of the cell surface molecule, gp130, which subsequently transduces a signal that activates intracytoplasmic Janus activated kinase (JAK) tyrosine kinase. JAK tyrosine kinase preferentially induces tyrosine phosphorylation of signal transducer and activator of transcription 3 (STAT3) [6]. In addition to roles of STAT3 in cell survival, growth, and differentiation [7], STAT3 is closely related to osteoclastogenesis [8]. RANKL, induced by the IL-6/sIL-6R complex, requires activation of STAT3 [8,9]. Although the roles of suppressor of cytokine signaling/cytokine-inducible SH2 (SOCS/CIS) have been retained, both SOCS1 and SOCS3 negatively regulate JAK tyrosine kinase as feedback inhibitors [6]. Shouda *et al*. demonstrated that inflammatory changes in joints and bone erosion were significantly suppressed in a collagen-induced arthritis animal model treated with SOCS-3 [10]. Therefore, regulation of STAT3 and SOCS3 in the FLS of patients with RA through the IL-6/gp130/STAT3 signaling pathway might be a potent therapeutic strategy in the treatment of RA.

Tacrolimus (FK506) is a macrolide immunosuppressant that primarily interferes with T cell activation and proliferation through inhibition of calcineurin, a calcium-dependent phosphatase that activates the nuclear factor of activated T cells (NFAT) transcription factor [11]. In addition to the anti-arthritic effects of tacrolimus through regulation of inflammatory cytokine production in RA [12,13], there is some evidence that tacrolimus may have a role in the regulation of bone metabolism. Tacrolimus prevents differentiation of these cells into mature osteoclasts through the calcineurin-NFAT pathway [14,15]. Tacrolimus was shown to have a protective effect on bone resorption in rats [16].

The blockade of RANKL expression in FLS may be important in the regulation of osteoclast differentiation for bone erosion in RA, because FLS is a potent source of RANKL production in patients with RA. In the current study, we investigated the potential roles of a calcineurin inhibitor, tacrolimus, in the regulation of RANKL expression through the IL-6-induced JAK-STAT signaling pathway in RA FLS.

## Methods

### Cell culture

Synoviocytes were isolated from the synovial tissues of four patients with RA (three women and one man) during total knee replacement surgery. Patients with RA met the American College of Rheumatology 1987 revised classification criteria for RA diagnosis [17]. Synovial tissues were harvested and incubated with collagenase type I (1 mg/ml) and hyaluronidase type I (2 mg/ml) for 2 hours at 37°C. After removing the large tissue, floating cells and synovial fibroblasts were isolated from adherent cells. Synovial fibroblasts were maintained in (D)MEM (Gibco, BRL, Grand Island, NY, USA) supplemented with 10% fetal bovine serum (Hyclone, Logan, UT, USA), 100 U/ml penicillin, and 100 μg/ml streptomycin. Subcultures were performed when cells reached 80% to 90% confluence. For the experiments, cells from passages three to eight were used. The protocol of this study was approved by the Institutional Review Board/Ethics Committee at the Catholic University of Daegu. Informed consent was obtained from the patients at the time of study enrollment.

### Viability assay

Cell viability was measured by the 3-(4,5-dimethylthiazol-2-yl)-2,5-diphenyltetra zolium bromide (MTT) assay (Sigma, St. Louis, MO, USA). Cells (2 × 10^4 ^cells/ml) were seeded in 96-well plates and incubated for 24 hours. Media were removed and cells were treated with different doses of drugs and incubated for 24 hours. An MTT (0.5 mg/ml) solution of 50 µl was added to each well. After incubation at 37°C for 4 hours, the MTT solution was removed and 100 µl of dimethyl sulfoxide (DMSO) was added. Cells were incubated at room temperature for an additional 10 minutes after which absorbance was measured at 540 nm with a plate reader (BMG Lab Technologies, Offenburg, Germany).

### Preparation of arthritis models and treatment

C57BL/6 mice (SLC Inc., Shizuoka, Japan) weighing 20 to 25 g at the beginning of the experiment were allocated to each study group, such as control mice (n = 6), mice treated with tacrolimus (n = 6), and mice not treated with tacrolimus (n = 6). K/BxN serum was provided by SI Lee (Gyeongsang National University School of Medicine, Jinju, Gyeongnam, Republic of Korea). We also appreciate the great contribution to this experiment of KRN TCR transgenic mice provided by D. Mathis and C. Benoist (Harvard Medical School, Boston, MA, USA) for the preparation of the K/BxN serum-induced arthritis. All experimental animals used in this study were maintained under the protocol approved by the Institutional Animal Care and Use Committee of the Gyeongsang National University (GLA-101116-M0112). Tacrolimus (1 mg/kg) was intraperitoneally injected into the mice four times a week. In the control group, normal saline was injected at the same frequency. C57BL/6 mice treated with/without tacrolimus subsequently received intraperitoneal injections of 150 μl of K/BxN serum. Following treatment, the mice were monitored daily for signs of arthritis. Ankle thickness was evaluated with a steel vernier caliper.

Histopathological scoring was performed on the knee joints of mice in each experimental group as previously described [18]. Six H & E-stained sections per each experimental animal were scored by two independent observers (K-Y Park and S-K Kim) at both low and high power fields. Scores ranged from 0 (normal) to a maximum of 5 (severe infiltration of inflammatory cells for inflammation, full thickness defect in the cortical bone, and marked trabecular bone loss for bone erosion).

### Quantitative real time-polymerase chain reaction (RT-PCR)

Cells were plated at a density of 2 × 10^6 ^cells per 100 mm on culture dishes and pretreated with 100 ng/ml IL-6/sIL-6R for 24 hours at 37°C. Various concentrations of tacrolimus (10, 100 and 1,000 nM) were then added to the culture for 24 hours at 37°C. Total RNA was extracted from the cells and the wrists sampled from sacrificed experimental mice using Trizol reagent (Gibco BRL, Grand Island, NY, USA). RNA was reverse transcribed to complementary DNA using the Improm-II Reverse Transcription System (Promega, Madison, WI, USA). A total of 1 μg RNA was mixed with Oligo(dT)_15 _primer (0.5 μg/μL; Promega) and heated to 70°C for 5 minutes and 4°C for 5 minutes. Reverse transcription was added to the 100U reaction buffer along with 0.5 mM deoxynucleoside triphosphate (dNTP), 4 mM MgCl_2_, 1 mM DTT, 5U Improm II reverse transcriptase, and 20 U recombinant ribonuclease inhibitor (RNasin). Nuclease free water was added in a final volume of 20 µL, and the reaction was annealed at 25°C for 5 minutes followed by extension at 42°C for 1 hour.

RT-PCR was performed using the Mini Option TM RT-PCR system (Bio-Rad, Hercules, CA, USA) with the DyNAmo SYBR Green qPCR kit (FINNZYMES, Espoo, Finland) according to the manufacturers' instructions. The reaction was performed in a total volume of 20 µL containing 10 µL of master mix, 10 pmol/L of each primer, 1 µL of cDNA, and 7 µL of distilled water. The following PCR protocols were used: 95°C for 3 minutes; 40 cycles (15 seconds, 95°C/1 minute, 60°C); and 72°C/45 seconds; and 60°C to 95°C per cycle for melting curve analysis.

RANKL primer sequences were forward 5′-GCT TGA AGC TCA GCC TTT TG-3′ and reverse 5′-CGA AAG CAA ATG TTG GCA TA-3′. Osteoprotegerin (OPG) primer sequences were forward 5′-GAA CCC CAG AGC GAA ATA CA-3′ and reverse 5′-TAT TCG CCA ACT GAG CA-3′. The β-actin primer sequences were forward 5′-CTG GAA CGG TGA AGG TGA CA-3′ and reverse 5′-AAG GGA CTT CCT GTA ACA CA-3′. Primers were synthesized by Bionics (Seoul, Korea). Data were analyzed with the delta delta Ct method.

### Western blot analyses

Cells were treated with 0, 30, 50, and 100 ng/ml IL-6/sIL-6R for 30 minutes. For another experiment, cells were treated with 100 ng/ml IL-6/sIL-6R for 30 minutes before the addition of one of two different concentrations of tacrolimus (0.5 or 1 µM). After incubation for 24 hours, cell (4 × 10^6^) pellets were lysed in a lysis buffer composed of 1 M Tris-HCl pH 8.0, 5 M NaCl, 10% Nonidet P40, and one tablet of protease inhibitor cocktail (Roche, Indianapolis, IN, USA). Cells were then incubated on ice for 10 minutes and centrifuged at 12,000 rpm for 10 minutes at 4°C. The pellet was discarded and the total protein concentration in the supernatant was determined using the Bio-Rad protein assay kit (Bio-Rad, Hercules, CA, USA). Proteins (30 to 60 μg) were separated by 10% SDS-PAGE gel electrophoresis, transferred to nitrocellulose membranes (Bio-Rad), and probed with appropriate antibodies. Antibodies to p-STAT3 (Y705), STAT3, and RANKL were obtained from Santa Cruz Biotechnology (Santa Cruz, CA, USA). Antibodies to p-JAK2 (Tyr1007/1008), JAK2, nuclear factor-κB (NF-κB), p-NF-κB, and NFAT were obtained from Cell Signaling Technology (Beverly, MA, USA). Antibodies to OPG and SOCS3 were purchased from Abcam (Cambridge, UK). Primary antibodies were incubated overnight at 4°C and horseradish peroxidase-conjugated secondary antibodies were incubated for 1 hour at room temperature. Proteins were detected with the SuperSignal^® ^West Pico chemiluminescent kit (Thermo Scientific, Rockford, IL, USA). Densitometry values were analyzed and quantified with Quantity One software (Bio-Rad).

### Transfection of siRNA

Cells were plated at approximately 80% confluence and transfected with siRNA via the lipofectamine^® ^RNAiMAX reagent (Invitrogen, Carlsbad, CA, USA). The siRNA for human SOCS3 and the Stealth™RNAi negative control were purchased from Invitrogen. SiRNA (50 nM) and lipofectamine^® ^RNAiMAX reagent in Opti-MEM (Invitrogen) were mixed and incubated at room temperature for 20 minutes. The mixtures were then added to each dish containing cells and incubated at 37°C for 72 hours. The transfected cells were treated with IL-6/sIL-6R at 100 ng/ml for 30 minutes.

### Enzyme-linked immunosorbent assay (ELISA)

A total of 2 × 10^4 ^cells were plated in 96-well culture plates. Cells were stimulated by IL-6/sIL-6R at 100 ng/ml for 24 hours followed by treatment with tacrolimus (0.01, 0.1 and 1 µM), methotrexate (MTX) (1 µg), and dexamethasone (1µg) for 24 hours at 37°C. RANKL and OPG were measured using ELISA Kits (Uscn Life Science Inc., Wuhan, China for RANKL and R&D Systems, Minneapolis, MN, USA for OPG) according to the manufacturers' instructions. ELISA plates with 96 wells (Nunc, Rochester, NY, USA) were coated with 2 µg/ml mouse monoclonal antihuman OPG and incubated overnight at room temperature. After washing the plates, recombinant human OPG standards and cell culture supernatants were added. The detection antibody, biotinylated polyclonal goat anti-human OPG at 200 ng/ml and streptavidin-HRP conjugate were added. The plates were washed again and hydrogen peroxide/tetramethylbenzidine substrate was added. The reaction was stopped and measured at 450 nm. Cell culture supernatants and human RANKL standards were added to pre-coated 96-well ELISA plates for 2 hours at 37°C. Detection color reagents A (H_2_O_2_) and B (TMB) were added for 1 hour, washed, and then reacted with substrate solution for 20 minutes. Stop solution was added to stop the reaction and absorbance was determined using a microplate reader at 450 nm.

### Immunofluorescence staining

Cells were seeded at a density of 5 × 10^4 ^cells on four-well glass slides (Nunc). The cells were fixed with 3.7% paraformaldehyde for 10 minutes at room temperature. Afterwards, the slides were washed twice with PBS and then blocked with 1% BSA in PBS for 30 minutes. Slides were incubated with primary antibody diluted in PBS for 1 hour. After washing with 0.1% Tween 20 in PBS, the slides were incubated with donkey anti-goat IgG-FITC or goat anti-rabbit IgG-FITC (Santa Cruz Biotechnology) for 40 minutes at room temperature in the dark. Cover slips were mounted onto the slide and slides were visualized by fluorescence microscopy (TE2000-U, Nikon Instruments Inc., NY, USA).

### Tartrate-resistant acid phosphatase (TRAP) staining

Peripheral blood mononuclear cells (PBMCs) were isolated from human blood obtained from three female RA patients by centrifugation using Histopaque^®^-1038 (Sigma-Aldrich, St. Louis, MO, USA) at 1800 rpm for 20 minutes at 4°C. Collected PBMCs (5 × 10^4 ^cells/well) were incubated in 96 well plates containing 60 ng/ml of RANKL and 50 ng/ml of M-CSF (Peprotech, East Brunswick, NJ, USA) in the presence or absence of tacrolimus. After 15 days, cells were fixed for 30 seconds and stained with TRAP staining kit (Sigma-Aldrich). Then, cells were incubated in a light protected incubator for 1hour at 37°C. Counterstain to Gill's hematoxylin solution was used for 2 minutes. TRAP-positive multinuclear cells were observed under a light microscope.

### Statistical analysis

Data are expressed as the mean ± standard deviation of three independent experiments. Statistical results were analyzed using the Mann-Whitney test. Data were analyzed using SPSS version 13.0 for Windows (SPSS Inc., Chicago, IL, USA). *P*-values less than 0.05 were considered statistically significant.

## Results

### Expression of IL-6/sIL-6R-induced RANKL and OPG in RA synoviocytes

**Figure 1:**
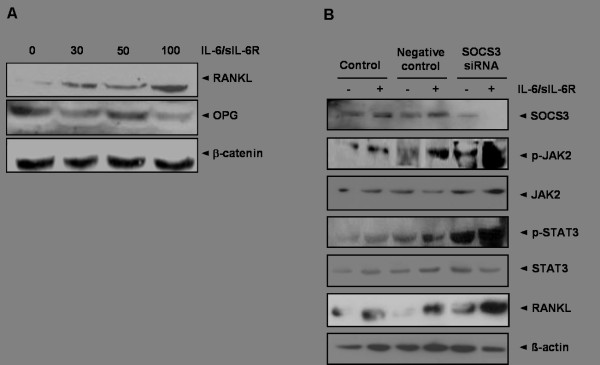
**The effect of the IL-6/sIL-6R complex on RANKL, JAK2, STAT3 in fibroblast-like synoviocytes (FLS)**. **(A) **Stimulation of FLS with IL-6 and sIL-6R at several different concentrations (0, 30, 50, 100 ng of both) for 30 minutes induced RANKL protein expression in a dose-dependent manner. In contrast, expression of OPG protein was gradually inhibited under the same conditions. **(B) **Treatment with IL-6/sIL-6R (100 ng of both) for 30 minutes in control synoviocytes increased the expression of SOCS3, p-JAK2, p-STAT3, and RANKL proteins. In SOCS3-siRNA transfected FLS, expression of p-JAK2, p-STAT3, and RANKL under IL-6/sIL-6R stimulation (100 ng/ml each) for 30 minutes was significantly increased compared to control synoviocytes. Data are determined in three independent experiments. IL-6, interleukin-6; JAK2, Janus activated kinase; OPG, osteoprotegerin; RANKL, receptor activator of NF-κB ligand; sIL-6R, soluble interleukin-6 receptor; SOCS3, suppressor of cytokine signaling 3; STAT3, signal transducer and activator of transcription-3.

RANKL and OPG are essential components in the regulation of osteoclastogenesis. OPG is known to be a soluble decoy receptor for RANKL, which functions to inhibit RANKL-RANK interaction as well as osteoclast maturation and activation. We found that IL-6/sIL-6R increased RANKL expression in a dose-dependent manner, whereas OPG expression after IL-6/sIL-6R treatment was decreased compared to untreated cells (Figure 1A).

As illustrated in Figure 1B, treatment of each 100 ng of IL-6/sIL-6R led to a prominent induction of p-JAK2 and p-STAT3. In addition, enhanced expression of SOCS3 and RANKL might be induced by activation of the JAK-STAT signaling pathway, which is stimulated by IL-6/sIL-6R. Stronger expression of p-JAK2, p-STAT3, and RANKL was detected in SOCS3 knockdown FLS using SOCS3 siRNA following IL-6/sIL-6R stimulation (Figure 1B).

### Inhibitory effects of tacrolimus on RANKL expression in a serum-induced arthritis model

**Figure 2:**
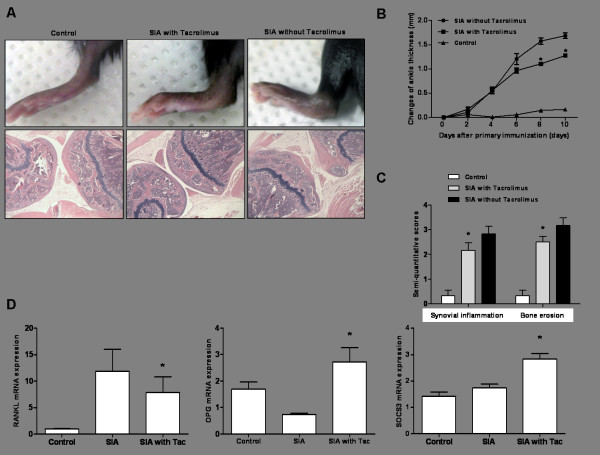
**The therapeutic effect of tacrolimus on inflammation and bone erosion in a serum-induced arthritis mouse model**. **(A) **Before immunization of C57BL/6 mice with K/BxN serum, tacrolimus (1 mg/kg) was intraperitoneally introduced four times for a week. At day 10, the experimental mice were sacrificed. Serum-induced arthritic mice pretreated with tacrolimus showed less paw swelling compared to those not treated with tacrolimus. Histological findings demonstrated protection against joint damage in the cartilage, bone, and synovium of tacrolimus-treated mouse joints. **(B) **Ankle thickness in the tacrolimus-treated mice was significantly less than that of non-treated serum-induced arthritis mice on days 8 and 10 (^*^P <0.05). **(C) **Semi-quantitative analysis for inflammation and bone erosion indicated fewer inflammatory changes and less bone destruction in tacrolimus-treated mice compared to untreated mice (^*^P <0.05). **(D) **Tacrolimus treatment significantly reduced RANKL mRNA expression in the affected joints of mice with serum-induced arthritis (^*^P <0.05). In contrast, the reduction in OPG mRNA expression in serum-induced arthritis was reversed with treatment by tacrolimus (^*^P <0.05). SOCS3 mRNA expression also was increased in arthritic joints treated with tacrolimus (^*^P <0.05 versus serum-induced arthritis not treated with tacrolimus). OPG, osteoprotegerin; RANKL, receptor activator of NF-κB ligand; SIA, serum-induced arthritis; SOCS3, suppressor of cytokine signaling 3; Tac, tacrolimus.

Arthritis was successfully induced after injection of K/BxN serum into C57B/L6 mice. Histological evaluations demonstrated that joint destruction was significantly attenuated in mice treated with tacrolimus compared to those not treated, as evidenced by enhanced inflammatory cell infiltration, cartilage abrasion, and bony erosion (Figure 2A). Compared to mice not treated with tacrolimus, mice treated with tacrolimus had significantly thinner ankles, a marker of joint inflammation, on day 8 and day 10 after primary immunization (*P *<0.05 on day 8 and *P *<0.05 on day 10) (Figure 2B). Semi-quantitative pathological analysis was performed on knee joints and showed that synovial inflammation and bony erosion were significantly reduced in tacrolimus-treated arthritic mice compared to mice not treated with tacrolimus (*P *<0.05 and *P *<0.05, respectively) (Figure 2C).

RANKL gene expression in affected wrist joints is prominently induced in serum-induced arthritis (Figure 2D). However, tacrolimus was found to decrease RANKL expression in the arthritis model compared to mice not treated with tacrolimus (*P *<0.05). In contrast, OPG gene expression in arthritic mice was more induced in tacrolimus-treated arthritis (*P *<0.05). These results indicate that tacrolimus is involved in osteoclastogenesis in inflammatory arthritis. In addition, tacrolimus significantly induced SOCS3 mRNA expression in affected joints of the arthritis model compared to the non-treated arthritic animals (*P *<0.05).

### Regulation of RANKL and OPG expression in the IL-6/sIL-6R-stimulated FLS by tacrolimus

**Figure 3:**
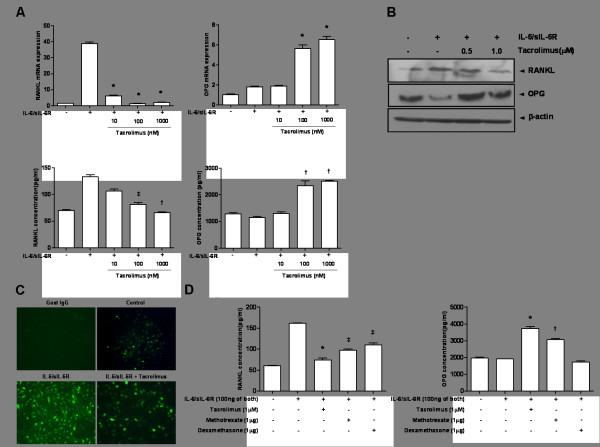
**Regulatory effect of tacrolimus on RANKL and OPG expression in FLS under IL-6/sIL-6R stimulation**. **(A) **After pretreatment of cultured FLS with IL-6/sIL-6R (100 ng of both), incubation with tacrolimus at several concentrations led to a consistently marked reduction of RANKL at the mRNA (^*^P <0.001 at 10, 100, 1,000 nM of tacrolimus) and protein levels (^‡^*P *<0.05 at 100 nM and ^†^*P *<0.01 1,000 nM of tacrolimus). In contrast, tacrolimus at dosages of 100 and 1,000 nM significantly increased levels of OPG mRNA (^*^P <0.001 for each dosage). Expression of OPG protein was consistently observed (^†^*P *<0.01 of 100 and 1,000 nM). **(B) **In western blot analysis for RANKL expression after tacrolimus treatment, tacrolimus was found to inhibit RANKL expression in FLS stimulated by IL-6/sIL-6R (100 ng of both). In contrast, OPG expression was increased following tacrolimus treatment. **(C) **Tacrolimus was shown to inhibit the expression of RANKL in FLS after stimulation with IL-6/sIL-6R in the immunofluorescence assay. **(D) **The inhibitory effect of tacrolimus (1 µM) on RANKL expression was more prominent than that of other anti-inflammatory drugs such as methotrexate (1 µg) and dexamethasone (1 µg). OPG mRNA expression was increased by tacrolimus and methotrexate but not by dexamethasone. (^*^P <0.001, ^†^*P *<0.01, ^‡^*P *<0.05 versus IL-6/sIL-6R-treated FLS). Data are determined in three independent experiments. FLS, fibroblast-like synociocytes; IL-6, interleukin-6; OPG, osteoprotegerin; RANKL, receptor activator of NF-κB ligand; sIL-6R, soluble interleukin-6 receptor.

Tacrolimus markedly suppressed RANKL mRNA expression in IL-6/sIL-6R-induced FLS (Figure 3A). In contrast, OPG expression in IL-6/sIL-6R-induced FLS was consistently increased at dosages of 100 and 1,000 nM of tacrolimus (*P *<0.001 for both). Treatment with tacrolimus reduced RANKL production in the supernatants of cells cultured under the same experimental conditions, whereas OPG concentrations were increased with tacrolimus treatment (Figure 3A).

Tacrolimus inhibited RANKL protein synthesis, whereas it enhanced the expression of OPG protein (Figure 3B). The presence of RANKL staining cells among cultured FLS was minimal in the immunofluorescence assay (Figure 3C). Treatment with tacrolimus significantly reduced the number of RANKL staining cells compared to FLS stimulated with IL-6/sIL-6R alone.

In addition, we compared the efficacy of tacrolimus in regulating RANKL and OPG expression to that of other drugs including MTX and dexamethasone (Figure 3D). All three experimental drugs showed inhibitory effects on RANKL protein production (*P *<0.001 for tacrolimus, *P *<0.05 for MTX, and *P *<0.05 for dexamethasone). Regarding effects on OPG expression, tacrolimus and MTX significantly enhanced OPG expression (*P *<0.001 for tacrolimus and *P *<0.01 for MTX), but dexamethasone did not (*P *>0.05).

### The effects of tacrolimus on the JAK-STAT-SOCS3 signaling pathway

**Figure 4:**
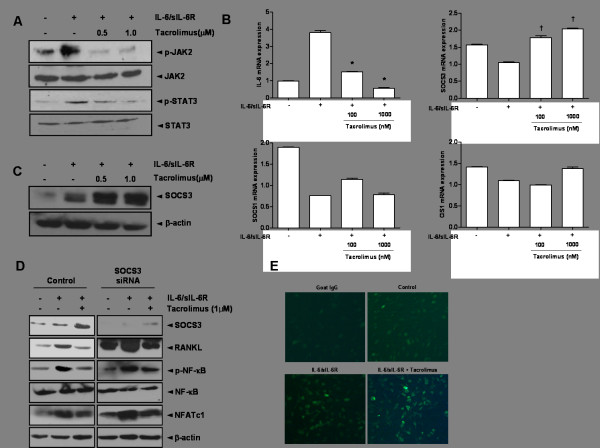
**The effect of tacrolimus on JAK2, STAT3, and SOCS3 in IL-6/sIL-6R-stimulated FLS**. **(A) **Stimulation with IL-6/sIL-6R (100 ng of both) induced phosphorylation of JAK2 and STAT3. However, tacrolimus reversed these changes, thereby significantly reducing the expression of p-JAK2 and p-STAT3. **(B) **Tacrolimus treatment of IL-6/sIL-6R-stimulated FLS potently suppressed IL-6 expression. Among negative regulators of the JAK-STAT signaling pathway, prominent induction of SOCS3 mRNA expression was induced by tacrolimus (^†^*P *<0.05 at 100 and 1,000 nM) in comparison to IL-6/sIL-6R-stimulated FLS. Expression of SOCS1 and CIS1 mRNA was not similarly induced. **(C) **Tacrolimus enhanced the level of SOCS3 protein in IL-6/sIL-6R-treated FLS in a dose-dependent manner. **(D) **In SOCS3 knockdown FLS, IL-6/sIL-6R induced overexpression of RANKL, p-NF-κB and NFATc1. In contrast, addition of tacrolimus induced SOCS3 expression and attenuated RANKL expression. The protein levels of p-NF-κB and NFATc1 were significantly reduced, in comparison to those in SOCS3 knockdown FLS without tacrolimus. **(E) **The immunofluorescence assay indicated the presence of an increased number of SOCS3-positive cells after treatment with tacrolimus compared to controls. Data are determined in three independent experiments. CIS1, cytokine-inducible SH2; IL-6, interleukin-6; JAK2, Janus activated kinase; RANKL, receptor activator of NF-κB ligand; sIL-6R, soluble interleukin-6 receptor; SOCS1, suppressor of cytokine signaling 1; SOCS3, suppressor of cytokine signaling 3; STAT3, signal transducer and activator of transcription 3.

Phosphorylation of JAK2 and STAT3 in IL-6/sIL-6R-stimulated FLS was significantly decreased by the addition of tacrolimus at doses of 0.5 and 1.0 µM (Figure 4A). Co-stimulation with IL-6/sIL-6R consistently reduced SOCS1, SOCS3 and CIS1 mRNA expression at the transcriptional level; however, IL-6 mRNA expression was increased (Figure 4B). Treatment with tacrolimus at both 100 and 1,000 nM dosages markedly enhanced SOCS3 mRNA expression (*P *<0.05 of both). However, both SOCS1 and CIS1 were not affected by tacrolimus treatment.

In the assessment of the effects of tacrolimus on the expression of RANKL and SOCS3, tacrolimus markedly increased the expression of the SOCS3 protein in a dose-dependent manner, as evidenced by western blot analysis (Figure 4C). Tacrolimus treatment (1 µM) in IL-6/sIL-6R-induced FLS enhanced SOCS protein expression, but significantly reduced expressions of RANKL and two transcription factors, the activated form of NF-κB and NFATc1 (Figure 4D). In SOCS3 knockdown FLS, overexpression of RANKL, p-NF-κB, and NFATc1 was seen under stimulation of IL-6/sIL-6R. In contrast, addition of tacrolimus in SOCS3-knockdown FLS significantly attenuated overexpressions of these molecules. This could suggest that enhanced SOCS3 expression by addition of tacrolimus contributed to the down-regulation of NF-κB and NFATc1 transcription factors in SOCS3 knockdown cells. Immunofluorescence studies also consistently demonstrated that tacrolimus increased the expression of SOCS3 in IL-6/sIL-6R-stimulated FLS (Figure 4E).

**Figure 5:**
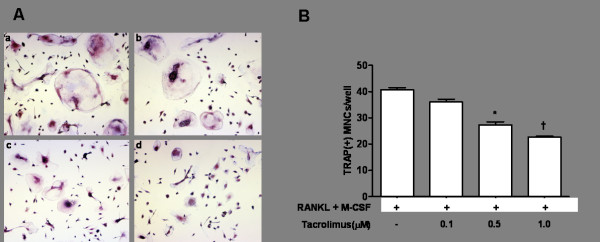
**The effect of tacrolimus on the formation of TRAP(+) multinucleated cells**. **(A) **TRAP staining assay showed that 60 ng/ml of RANKL and 50 ng/ml of M-CSF induced differentiation of PBMCs into TRAP (+) multinucleated cells, implicating osteoclasts (a). However, addition of tacrolimus gradually decreased the number of TRAP (+) multinucleated osteoclasts in a tacrolimus dose-dependent manner (b to d) (magnitude × 200). **(B) **Tacrolimus significantly suppressed number of TRAP(+) cells at 0.5 Mm and 1.0 Mm doses (^*^P <0.05 and ^†^*P *<0.01). M-CSF, macrophage colony-stimulating factor; RANKL, receptor activator of NF-κB ligand; TRAP, tartrate-resistant acid phosphatase.

The TRAP staining assay for osteoclasts using PBMC obtained from RA patients was performed to confirm the inhibitory effect of tacrolimus on osteoclast differentiation. Tacrolimus suppressed osteoclast differentiation in a dose-dependent manner, as illustrated in Figure 5A. The number of TRAP positive cells was significantly reduced after addition of 0.5 or 10 µM of tacrolimus (*P *<0.05 and *P *<0.01, respectively) (Figure 5B).

## Discussion

There is some evidence indicating that RANKL plays an important role as a regulator of osteoclastogenesis in the pathogenesis of RA [4]. It is well known that RANKL arises from osteoblast/stromal cells and activated T lymphocytes [1,19]. Pro-inflammatory cytokines including TNF-α, IL-17, and IL-1 are involved in the regulation of RANKL mRNA levels and proteins produced by FLS in mice and humans with RA [3-5]. Two previous studies reported the induction of RANKL by TNF-α, IL-17, and IL-1β in RA FLS [3,5]. Hashizume *et al*. demonstrated that both TNF-α and IL-17 increased RANKL expression only in association with sIL-6R [4]. Furthermore, they showed that IL-6 also stimulated RANKL expression in FLS in the presence of sIL-6R. In this study, co-treatment of FLS with IL-6 and sIL-6R significantly increased the protein and mRNA levels of RANKL. This suggests that activation of the IL-6 trans-signaling pathway might trigger osteoclastogenesis through enhanced RANKL expression in FLS of subjects with RA.

IL-6 binding to sIL-6R activates JAK tyrosine kinase and STAT transcriptional factor. Because its tyrosine phosphorylation was detected exclusively in synovial tissues of RA but not those of osteoarthritis, STAT3 is considered a crucial molecule in the pathogenesis of RA [10]. The IL-6/sIL-6R-treated stromal/osteoblastic cell line (UAMS-32) with dominant negative-STAT3 protein was blocked to induce RANKL expression [9]. These findings suggest that the regulation of STAT3 is critical for the control of osteoclastogenesis by activation of gp-130-mediated cytokines. Treatment of IL-6/sIL-6R-stimulated FLS with parthenolide, a STAT inhibitor, reduced the expression of RANKL mRNA [4]. Therefore, STAT3 activation is essential for transcription in osteoclastogenesis through regulation of RANKL expression in the IL-6/sIL-6R-activated signaling pathway. SOCS molecules, a family of eight different intracellular proteins, were first identified as negative feedback factors for cytokine-related responses [20]. Now, SOCS proteins are considered important players in the regulation of the cytokine-JAK-STAT signaling pathway. Both SOCS1 and SOCS3 have been identified as potential inhibitors of JAK tyrosine kinase activity [6]. There is some evidence that SOCS3 is a crucial negative regulator of IL-6 signaling. Prolonged phosphorylation in SOCS3 gene-deficient mouse macrophages due to stimulation with IL-6 suggests that SOCS3 plays an important role in controlling the responses to IL-6 [21]. In the present study, we found that the IL-6/sIL-6R complex in cultured RA synoviocytes led to phosphorylation of JAK2 and STAT3 molecules. In addition, the expression of the SOCS3 protein was markedly increased after stimulation with IL-6/sIL-6R. Furthermore, the IL-6/sIL-6R complex resulted in increased phosphorylation of both JAK2 and STAT3, as well as increased RANKL protein expression in SOCS3 siRNA-transfected RA FLS compared to control FLS. Our data suggest that RANKL expression in FLS treated with IL-6/sIL-6R might be primarily dependent on the JAK2-STAT3-SOCS3 signaling pathway.

Tacrolimus is a potent immunosuppressive drug. It primarily plays a role in the inhibition of T cell activation by targeting a calcium-dependent calcineurin phosphatase of the NFAT transcription factor [11]. Tacrolimus reduced the number of TRAP-positive human mononuclear cells (MNCs) expressing RANKL and M-CSF as well as the formation of lacunar resorption pits in a previous study [15]. Tacrolimus has a potent inhibitory effect on osteoclast differentiation. Inspection of rat upper maxilla treated with tacrolimus (1 mg/kg/day) for 60 days demonstrated an increase in alveolar bone volume secondary to a decrease in osteoclast number compared to rats treated with a drug vehicle [22]. Another study suggested that the anti-osteoclastic effect of tacrolimus might be explained by its induction of apoptosis in osteoclasts [23].

However, data about the effect of tacrolimus on RANKL expression in RA synoviocytes has not been identified. Our study showed that tacrolimus inhibits bone erosion in a serum-induced arthritis mouse model, compared to serum-induced arthritis mice not treated with tacrolimus. The effect on bone erosion was seen in addition to the anti-inflammatory effect of tacrolimus on synovial inflammation in arthritis. The mRNA levels of RANKL measured in the ankles of serum-induced arthritis models treated with tacrolimus were significantly lower than those not treated with tacrolimus. This result was confirmed by an *in vitro *experiment using RA FLS treated with IL-6/sIL-6R. These findings suggest that the protective role of tacrolimus against bone erosion is related to the reduction of RANKL production in tacrolimus-treated mice.

Inhibition of either STAT or JAK is considered an important therapeutic target to prevent bone destruction in RA [8,9,24]. The Pan-JAK inhibitor, pyridine 6, significantly suppressed osteoclast differentiation and bone resorption by inhibiting RANKL-induced NFATc1 expression in mouse bone marrow macrophage cultures [24]. In an experiment using STAT3 knockout mice, induction of RANKL was inhibited by stimulation with IL-6 and IL-6R [9]. Recently Mori *et al*. provide evidence that suppression of STAT3 might be beneficial by inhibiting osteoclatogenesis mediated by the IL-6/STAT3-dependent inflammatory cascade [8]. We investigated whether tacrolimus has an inhibitory effect on RANKL production by blocking or attenuating JAK2 and STAT3 activity in cultured RA synoviocytes treated with IL-6/sIL-6R. We observed that tacrolimus has inhibitory effects on the phosphorylation of both JAK2 and STAT3 in FLS stimulated with IL-6/sIL-6R. Our results suggest that tacrolimus may be involved in the activation of JAK-STAT signaling in RA synoviocytes. Furthermore, we demonstrated that down-regulation of JAK-STAT activation secondarily induced the expression of SOCS3, a negative regulator of STAT, whereas the expression of SOCS1 and CIS1 was not similarly induced. Functional SOCS1 deficiency is mainly involved in an unregulated response of IFN-γ, resulting in neonatal defects in SOCS^-/- ^mice [25]. The phenotypes of CIS transgenic mice are remarkably similar to those found in STAT5 KO mice, suggesting that CIS is an important regulator of STAT5-mediated cytokine responses [26]. However, SOCS3 is considered a crucial determinant of IL-6 signaling through negative feedback. This study also revealed that tacrolimus, a known inhibitor of JAK2 and STAT3 phosphorylation, increased SOCS3 expression in IL-6/sIL-6R-treated FLS.

The intracellular signaling pathways of RANKL-RANK are mediated by activation of several crucial transcription factors including NF-κB and NFATc1 via TNF receptor-associated factor-6 (TRAF-6) during osteoclastogenesis [27]. In this study, we suggest that overexpression of NF-κB and NFATc1 in SOCS3 knockdown FLS was suppressed by enhanced SOCS3 expression through treatment with tacrolimus. Although tacrolimus could directly inhibit activation of NFATc1, Banerjee *et al*. showed that SOCS3 interacted with calcineurin and then suppressed the activation of NFAT in primary T cells [28]. Considering the effect of SOCS3 on activation of NF-κB, SOCS3 inhibited IL-1-mediated NF-κB activation through suppression of ubiquitination of TRAF-6 [29]. Based on this evidence, SOCS3 could play a role as a crucial regulator of both NF-κB and NFATc1 transcription factors.

Among several disease modifying anti-rheumatic drugs for RA, MTX demonstrates marked potency as an inhibitor of persistent synovial inflammation. Female Sprague-Dawley rats treated with intraperitoneal MTX injections exhibited a significant increase in urinary hydroxyproline, a marker of bone resorption [30]. These results suggest that bone metabolism in MTX-treated subjects is related to the upregulation of osteoclast activity. In contrast, *in vitro*, MTX therapy was shown to decrease the RANKL:OPG ratio in cultured osteoblasts [31]. In the present study, we assessed the inhibitory effect of RANKL expression and discovered that MTX (100 nM) has an inhibitory effect on RANKL production in IL-6-stimulated RA synoviocytes. The influence of dexamethasone on RANKL expression has been reported in different cell lines [32,33]. Our study demonstrated that dexamethasone (1,000 nM) decreased RANKL production in RA synoviocytes cultured with IL-6/sIL-6R. Although the differential effect of dexamethasone on RANKL remained, its effect on RANKL production in synoviocytes might be distinct from that in other osteoblastic or osteoclastic cells.

## Conclusions

**Figure 6:**
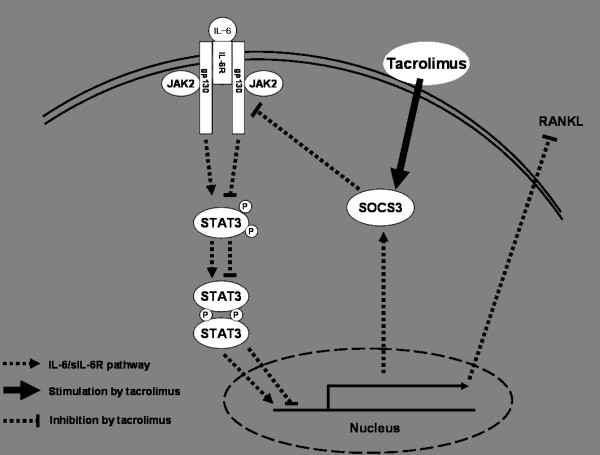
**Summary for the effect of calcineurin inhibitor, tacrolimus, on the regulation of RANKL expression through IL-6/sIL-6R/JAK2/STAT3/SOCS3 pathway**. IL-6, interleukin-6; JAK2, Janus activated kinase; RANKL, receptor activator of NF-κB ligand; sIL-6R, soluble interleukin-6 receptor; SOCS3, suppressor of cytokine signaling 3; STAT3, signal transducer and activator of transcription 3.

In summary, the cytokine IL-6, together with sIL-6R, has a pathogenic role in the development of RA through its effects on synovial inflammation and bone destruction. As such, it is considered a promising therapeutic target molecule. The intimate interaction between synoviocytes and osteoclasts contributes to the development of bone erosion. RANKL has an essential role in the regulation of osteoclast activation and differentiation. Our study showed that FLS is another source of RANKL production in synovial inflammation seen in RA. In addition, we found that RANKL expression by RA FLS depends on the JAK2-STAT3-SOCS3 signaling pathway at both the mRNA and protein levels. As shown in Figure 6, taken together these results indicate that tacrolimus has an inhibitory effect on RANKL expression in RA synoviocytes in both *in vivo *and *in vitro *experiments through its regulation of the JAK2-STAT3-SOCS3 pathway.

## Abbreviations

CIS: cytokine-inducible SH2; (D)MEM: (Dulbecco's) modified Eagle's medium; DMSO: dimethyl sulfoxide; ELISA: enzyme-linked immunosorbent assay; FLS: fibroblast-like synoviocytes; H & E: hematoxylin and eosin; IL: interleukin; IFN: interferon; JAK2: Janus activated kinase; M-CSF: macrophage colony-stimulating factor; MNCs: mononuclear cells; MTX: methotrexate; MTT: 3-(4,5-dimethylthiazol-2-yl)-2,5-diphenyltetra zolium bromide; NFAT: nuclear factor of activated T cells; NF-κB: nuclear factor-κB; OPG: osteoprotegerin; PBMC: peripheral blood mononuclear cells; PBS: phosphate-buffered saline; RA: rheumatoid arthritis; RANKL: receptor activator of NF-κB ligand; RNasin: ribonuclease inhibitor; RT-PCR: real time-polymerase chain reaction; sIL-6R: soluble IL-6 receptor; siRNA: small interfering RNA; SOCS3: suppressor of cytokine signaling 3; STAT3: signal transducer and activator of transcription 3; TNF: tumor necrosis factor; TRAP: tartrate-resistant acid phosphatase.

## Competing interests

The authors declare that they have no competing interests.

## Authors' contributions

SK was involved in the study concept and design. JY, SH and SK contributed to the acquisition and interpretation of data. KY and SI performed the animal experiments and molecular assays. All authors read and approved the final manuscript.

## References

[B1] Jimi E Akiyama S Tsurukai T Okahashi N Kobayashi K Udagawa N Nishihara T Takahashi N Suda T Osteoclast differentiation factor acts as a multifunctional regulator in murine osteoclast differentiation and function J Immunol 1999 163 434 442 10384146

[B2] Crotti TN Smith MD Weedon H Ahern MJ Findlay DM Kraan M Tak PP Haynes DR Receptor activator NF-κB ligand (RANKL) expression in synovial tissue from patients with rheumatoid arthritis, spondyloarthropathy, osteoarthritis, and from normal patients: semiquantitative and quantitative analysis Ann Rheum Dis 2002 61 1047 1054 10.1136/ard.61.12.1047 12429533 PMC1753975

[B3] Ainola M Mandelin J Liljeström M Konttinen YT Salo J Imbalanced expression of RANKL and osteoprotegerin mRNA in pannus tissue of rheumatoid arthritis Clin Exp Rheumatol 2008 26 240 246 18565244

[B4] Hashizume M Hayakawa N Mihara M IL-6 trans-signalling directly induces RANKL on fibroblast-like synovial cells and is involved in RANKL induction by TNF-α and IL-17. Rheumatology (Oxford) 2008 47 1635 1640 10.1093/rheumatology/ken363 18786965

[B5] Tunyogi-Csapo M Kis-Toth K Radacs M Farkas B Jacobs JJ Finnegan A Mikecz K Glant TT Cytokine-controlled RANKL and osteoprotegerin expression by human and mouse synovial fibroblasts: fibroblast-mediated pathologic bone resorption Arthritis Rheum 2008 58 2397 2408 10.1002/art.23653 18668542

[B6] Yoshimura A Nishinakamura H Matsumura Y Hanada T Negative regulation of cytokine signaling and immune responses by SOCS proteins Arthritis Res Ther 2005 7 100 110 10.1186/ar1741 15899058 PMC1174965

[B7] Hirano T Ishihara K Hibi M Roles of STAT3 in mediating the cell growth, differentiation and survival signals relayed through the IL-6 family of cytokine receptors Oncogene 2000 19 2548 2556 10.1038/sj.onc.1203551 10851053

[B8] Mori T Miyamoto T Yoshida H Asakawa M Kawasumi M Kobayashi T Morioka H Chiba K Toyama Y Yoshimura A IL-1β and TNFα-initiated IL-6-STAT3 pathway is critical in mediating inflammatory cytokines and RANKL expression in inflammatory arthritis Int Immunol 2011 23 701 712 10.1093/intimm/dxr077 21937456

[B9] O'Brien CA Gubrij I Lin SC Saylors RL Manolagas SC STAT3 activation in stromal/osteoblastic cells is required for induction of the receptor activator of NF-κB ligand and stimulation of osteoclastogenesis by gp130-utilizing cytokines or interleukin-1 but not 1,25-dihydroxyvitamin D3 or parathyroid hormone J Biol Chem 1999 274 19301 19308 10.1074/jbc.274.27.19301 10383440

[B10] Shouda T Yoshida T Hanada T Wakioka T Oishi M Miyoshi K Komiya S Kosai K Hanakawa Y Hashimoto K Nagata K Yoshimura A Induction of the cytokine signal regulator SOCS3/CIS3 as a therapeutic strategy for treating inflammatory arthritis J Clin Invest 2001 108 1781 1788 11748261 10.1172/JCI13568 PMC209467

[B11] Scott LJ McKeage K Keam SJ Plosker GL Tacrolimus: a further update of its use in the management of organ transplantation Drugs 2003 63 1247 1297 10.2165/00003495-200363120-00006 12790696

[B12] Magari K Miyata S Nishigaki F Ohkubo Y Mutoh S Goto T Differential effects of FK506 and methotrexate on inflammatory cytokine levels in rat adjuvant-induced arthritis J Rheumatol 2003 30 2193 2200 14528517

[B13] Magari K Miyata S Nishigaki F Ohkubo Y Mutoh S Comparison of anti-arthritic properties of leflunomide with methotrexate and FK506: effect on T cell activation-induced inflammatory cytokine production in vitro and rat adjuvant-induced arthritis Inflamm Res 2004 53 544 550 10.1007/s00011-004-1294-9 15597149

[B14] Hirotani H Tuohy NA Woo JT Stern PH Clipstone NA The calcineurin/nuclear factor of activated T cells signaling pathway regulates osteoclastogenesis in RAW264.7 cells J Biol Chem 2004 279 13984 13992 10.1074/jbc.M213067200 14722106

[B15] Miyazaki M Fujikawa Y Takita C Tsumura H Tacrolimus and cyclosporine A inhibit human osteoclast formation via targeting the calcineurin-dependent NFAT pathway and an activation pathway for c-Jun or MITF in rheumatoid arthritis Clin Rheumatol 2007 26 231 239 16586042 10.1007/s10067-006-0287-1

[B16] Spolidorio LC Nassar PO Nassar CA Spolidorio DM Muscará MN Conversion of immunosuppressive monotherapy from cyclosporin A to tacrolimus reverses bone loss in rats Calcif Tissue Int 2007 81 114 123 10.1007/s00223-007-9040-2 17612778

[B17] Arnett FC Edworthy SM Bloch DA McShane DJ Fries JF Cooper NS Healey LA Kaplan SR Liang MH Luthra HS The American Rheumatism Association 1987 revised criteria for the classification of rheumatoid arthritis Arthritis Rheum 1988 31 315 324 10.1002/art.1780310302 3358796

[B18] Pettit AR Ji H von Stechow D Müller R Goldring SR Choi Y Benoist C Gravallese EM TRANCE/RANKL knockout mice are protected from bone erosion in a serum transfer model of arthritis Am J Pathol 2001 159 1689 1699 10.1016/S0002-9440(10)63016-7 11696430 PMC1867076

[B19] Yasuda H Shima N Nakagawa N Yamaguchi K Kinosaki M Mochizuki S Tomoyasu A Yano K Goto M Murakami A Tsuda E Morinaga T Higashio K Udagawa N Takahashi N Suda T Osteoclast differentiation factor is a ligand for osteoprotegerin/osteoclastogenesis-inhibitory factor and is identical to TRANCE/RANKL Proc Natl Acad Sci USA 1998 95 3597 3602 10.1073/pnas.95.7.3597 9520411 PMC19881

[B20] Yoshimura A Ohkubo T Kiguchi T Jenkins NA Gilbert DJ Copeland NG Hara T Miyajima A A novel cytokine-inducible gene CIS encodes an SH2-containing protein that binds to tyrosine-phosphorylated interleukin 3 and erythropoietin receptors EMBO J 1995 14 2816 2826 7796808 10.1002/j.1460-2075.1995.tb07281.x PMC398400

[B21] Lang R Pauleau AL Parganas E Takahashi Y Mages J Ihle JN Rutschman R Murray PJ SOCS3 regulates the plasticity of gp130 signaling Nat Immunol 2003 4 546 550 10.1038/ni932 12754506

[B22] Andia DC Nassar CA Nassar PO Guimarães MR Cerri PS Spolidorio LC Treatment with tacrolimus enhances alveolar bone formation and decreases osteoclast number in the maxillae: a histomorphometric and ultrastructural study in rats Histol Histopathol 2008 23 1177 1184 18712669 10.14670/HH-23.1177

[B23] Igarashi K Hirotani H Woo JT Stern PH Cyclosporine A and FK506 induce osteoclast apoptosis in mouse bone marrow cell cultures Bone 2004 35 47 56 10.1016/j.bone.2004.02.009 15207740

[B24] Kwak HB Kim HS Lee MS Kim KJ Choi EY Choi MK Kim JJ Cho HJ Kim JW Bae JM Kim YK Park BH Ha H Chun CH Oh J Pyridone 6, a pan-Janus-activated kinase inhibitor, suppresses osteoclast formation and bone resorption through down-regulation of receptor activator of nuclear factor-κB (NF-κB) ligand (RANKL)-induced c-Fos and nuclear factor of activated T cells (NFAT) c1 expression Biol Pharm Bull 2009 32 45 50 10.1248/bpb.32.45 19122279

[B25] Alexander WS Starr R Fenner JE Scott CL Handman E Sprigg NS Corbin JE Cornish AL Darwiche R Owczarek CM Kay TW Nicola NA Hertzog PJ Metcalf D Hilton DJ SOCS1 is a critical inhibitor of interferon gamma signaling and prevents the potentially fatal neonatal actions of this cytokine Cell 1999 98 597 608 10.1016/S0092-8674(00)80047-1 10490099

[B26] Matsumoto A Seki Y Kubo M Ohtsuka S Suzuki A Hayashi I Tsuji K Nakahata T Okabe M Yamada S Yoshimura A Suppression of STAT5 functions in liver, mammary glands, and T cells in cytokine-inducible SH2-containing protein 1 transgenic mice Mol Cell Biol 1999 19 6396 6407 10454585 10.1128/mcb.19.9.6396 PMC84609

[B27] Edwards JR Mundy GR Advances in osteoclast biology: old findings and new insights from mouse models Nat Rev Rheumatol 2011 7 235 243 10.1038/nrrheum.2011.23 21386794

[B28] Banerjee A Banks AS Nawijn MC Chen XP Rothman PB Cutting edge: Suppressor of cytokine signaling 3 inhibits activation of NFATp J Immunol 2002 168 4277 4281 11970967 10.4049/jimmunol.168.9.4277

[B29] Frobøse H Rønn SG Heding PE Mendoza H Cohen P Mandrup-Poulsen T Billestrup N Suppressor of cytokine signaling-3 inhibits interleukin-1 signaling by targeting the TRAF-6/TAK1 complex Mol Endocrinol 2006 20 1587 1596 16543409 10.1210/me.2005-0301

[B30] May KP West SG McDermott MT Huffer WE The effect of low-dose methotrexate on bone metabolism and histomorphometry in rats Arthritis Rheum 1994 37 201 206 10.1002/art.1780370208 8129775

[B31] Revu S Neregård P af Klint E Catrina AI Methothrexate directly inhibits RANKL expression and osteoclast formation in very early arthritis [abstract] Ann Rheum Dis 2010 69 A23

[B32] Atkins GJ Kostakis P Pan B Farrugia A Gronthos S Evdokiou A Harrison K Findlay DM Zannettino AC RANKL expression is related to the differentiation state of human osteoblasts J Bone Miner Res 2003 18 1088 1098 10.1359/jbmr.2003.18.6.1088 12817763

[B33] Wei X Zhang X Zuscik MJ Drissi MH Schwarz EM O'Keefe RJ Fibroblasts express RANKL and support osteoclastogenesis in a COX-2-dependent manner after stimulation with titanium particles J Bone Miner Res 2005 20 1136 1148 10.1359/JBMR.050206 15940366

